# Two novel XRE-like transcriptional regulators control phenotypic heterogeneity in *Photorhabdus luminescens* cell populations

**DOI:** 10.1186/s12866-021-02116-2

**Published:** 2021-02-25

**Authors:** Simone Eckstein, Jannis Brehm, Michael Seidel, Mats Lechtenfeld, Ralf Heermann

**Affiliations:** 1 Johannes-Gutenberg-Universität Mainz, Institut für Molekulare Physiologie, Biozentrum II, Mikrobiologie und Weinforschung, Hanns-Dieter-Hüsch-Weg 17, 55128 Mainz, Germany; 2 Ludwig-Maximilians-Universität München, Biozentrum, Bereich Mikrobiologie, Martinsried, Germany

**Keywords:** Entomopathogenic bacteria, Phenotypic heterogeneity, Phenotypic switching, XRE-like regulators, Toxin/antitoxin-system (TAS)

## Abstract

**Background:**

The insect pathogenic bacterium *Photorhabdus luminescens* exists in two phenotypically different forms, designated as primary (1°) and secondary (2°) cells. Upon yet unknown environmental stimuli up to 50% of the 1° cells convert to 2° cells. Among others, one important difference between the phenotypic forms is that 2° cells are unable to live in symbiosis with their partner nematodes, and therefore are not able to re-associate with them. As 100% switching of 1° to 2° cells of the population would lead to a break-down of the bacteria’s life cycle the switching process must be tightly controlled. However, the regulation mechanism of phenotypic switching is still puzzling.

**Results:**

Here we describe two novel XRE family transcriptional regulators, XreR1 and XreR2, that play a major role in the phenotypic switching process of *P. luminescens*. Deletion of *xreR1* in 1° or *xreR2* in 2° cells as well as insertion of extra copies of *xreR1* into 2° or x*reR2* into 1° cells, respectively, induced the opposite phenotype in either 1° or 2° cells. Furthermore, both regulators specifically bind to different promoter regions putatively fulfilling a positive autoregulation. We found initial evidence that XreR1 and XreR2 constitute an epigenetic switch, whereby XreR1 represses *xreR2* expression and XreR2 self-reinforces its own gene by binding to XreR1.

**Conclusion:**

Regulation of gene expression by the two novel XRE-type regulators XreR1 and XreR2 as well as their interplay represents a major regulatory process in phenotypic switching of *P. luminescens*. A fine-tuning balance between both regulators might therefore define the fate of single cells to convert from the 1° to the 2° phenotype.

## Background

*Photorhabdus luminescens* subsp. *laumondii* DJC is a Gram-negative, entomopathogenic bacterium of the family of *Enterobacteriaceae* [1, 2]. *P. luminescens* harbors a complex dualistic life cycle including two hosts. Initially, the bacteria live in mutualistic symbiosis with infective juvenile (IJ) *Heterorhabditidae* nematodes colonizing their upper gut. These nematodes invade insect larvae such as *Galleria mellonella* where *P. luminescens* is released into the hemolymph to finally kill the insect [1]. The bacteria exist in two phenotypically different cell forms referred to as primary (1°) and the secondary (2°) cells [3]. During insect infection or during prolonged cultivation, a large portion of up to 50% of the 1° cells convert to the 2° form. The two cell forms are easy to distinguish as 1° cells exhibit specific phenotypic features that are absent in 2° cells. These properties include the biosynthesis of secondary metabolites like antibiotics or production of anthraquinones, which results in reddish-brown pigmentation, as well as bioluminescence or the formation of crystalline inclusion proteins, cell clumps and mucoid colony morphology [3–7]. Importantly, while both cell forms are equally pathogenic towards insects, 2° cells are not able to re-associate with the nematodes after depletion of nutrients derived by the insect host [7, 8]. Recently, 2° cells have been shown to specifically interact with plant roots and adapt to an alternative life-style when remaining in soil after the nematodes have left the depleted insect cadaver [9]. Since phenotypic switching from 1° to 2° cells also takes place after prolonged cultivation under laboratory conditions, a response to metabolic or environmental stress has been suggested [10]. So far, the switch has only been observed unidirectional occurring from 1° to 2° cells suggesting that a key signal which is missing under laboratory conditions [11].

Due to the deficiency of *P. luminescens* 2° to re-associate with the nematodes, phenotypic switching of the whole cell population would lead to a breakdown of the bacteria’s life cycle with respect to nematode symbiosis after insect infection. Therefore, the switching process has to be tightly controlled. To the current state of knowledge at least two pathways are suggested to be involved in controlling phenotypic switching: a HexA-dependent pathway and an O_2_-dependent pathway via the AstS/AstR tow-component system. HexA is a LysR-type transcriptional regulator which has been shown to suppress 1°-specific features in a versatile way, directly or indirectly [6, 12]. In contrast, AstS/AstR reacts to global stress signals and was shown to delay phenotypic switching. Although both systems seem to be activated by global stress factors, no direct connection between the two regulation pathways is known so far [10]. However, the complex regulatory network of phenotypic switching has not been fully understood yet [13]. The nematode-bacteria complexes are used in agricultural industry where they are cultivated in liquid media and then spread onto fields to prevent crop failure caused by insects. Hereby, the nematodes are pre-incubated with the bacterial symbiont as they essentially support their development and reproduction. Thus, phenotypic switching is one of the major reasons for process failure in industrial mass production [8] and therefore understanding of the phenotypic switching regulatory network is of high importance for the biotechnological applicability of entomopathogenic nematodes.

Recently, comparative transcriptome analysis of 1° and 2° cells was performed, which suggested putative further regulatory proteins involved in the switching process [7]. Thus, in total up about 640 genes were found to be differentially expressed in 2° cells. Among these some predicted regulators with yet unknown function were either highly up- or down-regulated in 2° cells [7]. It has been demonstrated that two of these transcriptional regulators, *PluDJC_21240* (XRE-transcriptional Regulator, *xreR2*) is up-regulated in 2° cells and *PluDJC_21265* (XRE-transcriptional Regulator *xreR1*) is up-regulated in 1° cells. XreR1 and XreR2 are two of in total 27 putative XRE-like regulators present in *P. luminescens* DJC. Here we show that XreR1 and XreR2 play an important role in the control of phenotypic switching in *P. luminescens*. Deletion or insertion of either *xreR2* or *xrerR1* in 1° as well as 2° cells, respectively, was sufficient to induce the respective opposite phenotype in either 1° or 2° cells. Furthermore, we could show a direct interaction of the regulators with DNA and we identified promoter regions to that both, XreR2 and XreR1, specifically bind to. Lastly, we found first evidence that XreR1 and XreR2 constitute an epigenetic switch whereby the 2° phenotype is maintained by high *xreR2* levels.

## Results

### Effect of XreR1 and XreR2 on 1°- and 2°-specific phenotypes of *P. luminescens*

**Fig. 1:**
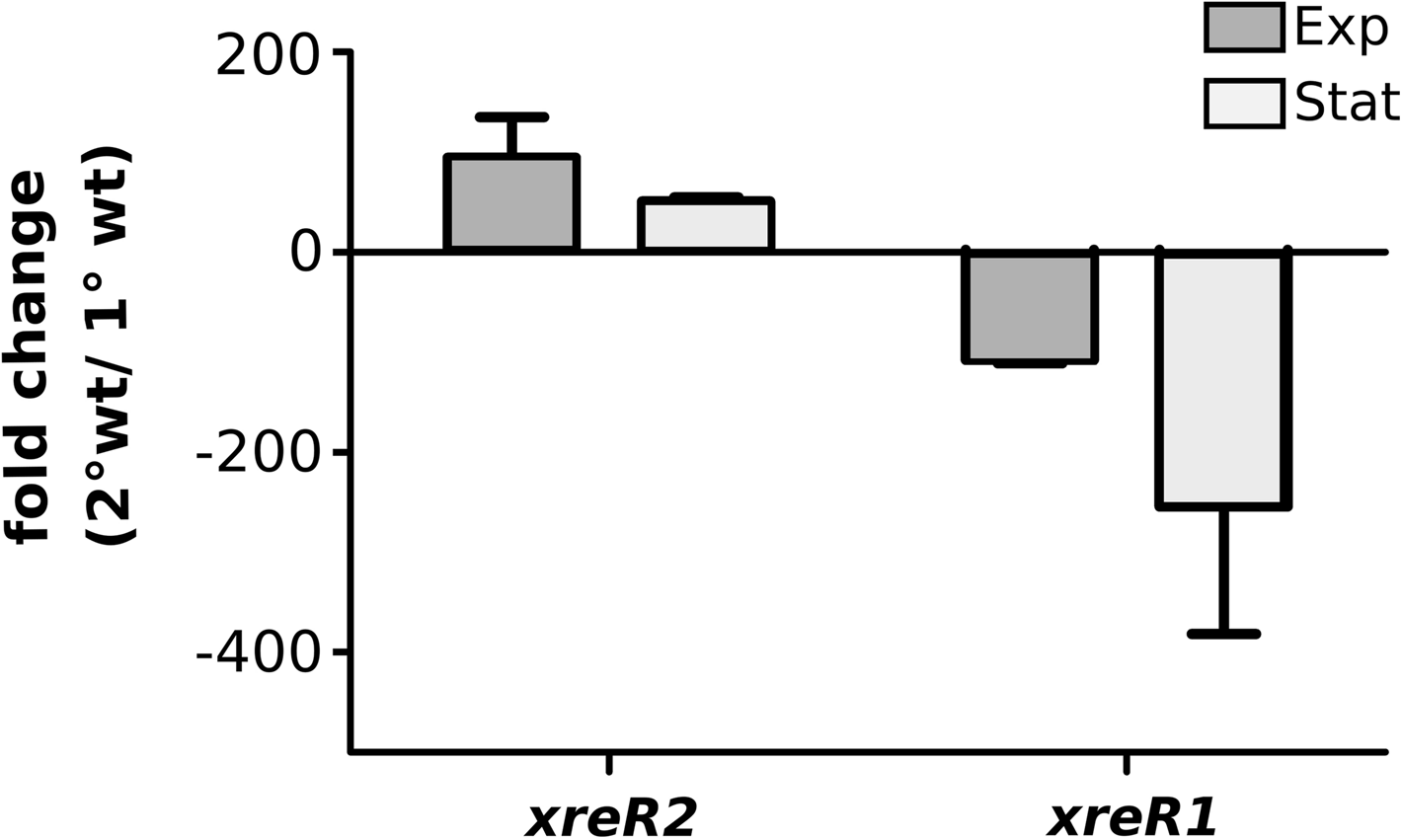
Expression of *xreR2* and *xreR1* in *P. luminescens* 1° and 2° cells. Gene expression levels of *xreR1* and *xreR2* in 1° as well as 2° cells was quantified via qRT-PCR. For that purpose, RNA was collected from the respective *P. luminescens* strains during exponential (Exp, dark grey bars) as well as stationary (Stat, light grey bars) growth phase via P/C/I extraction and gene expression was depicted comparatively (fold change) from 2° to 1° cells. Error bars represent standard deviation of three independently performed experiments

The high differences in expression of *xreR1* and *xreR2* in 2° compared to 1° cells observed in the transcriptome comparison [7] indicated an importance of those two transcriptional regulators in the process of phenotypic switching of *P. luminescens*. First, we verified an up-regulation of *xreR2* in 2° cells and higher transcription of *xreR1* in 1° cells via RT-qPCR. Whereas *xreR2* was upregulated approximately 100-fold in the exponential growth phase of 2° cells, xreR1 was upregulated approximately 300-fold in the stationary growth phase of 1° cells (Fig. 1).

**Fig. 2:**
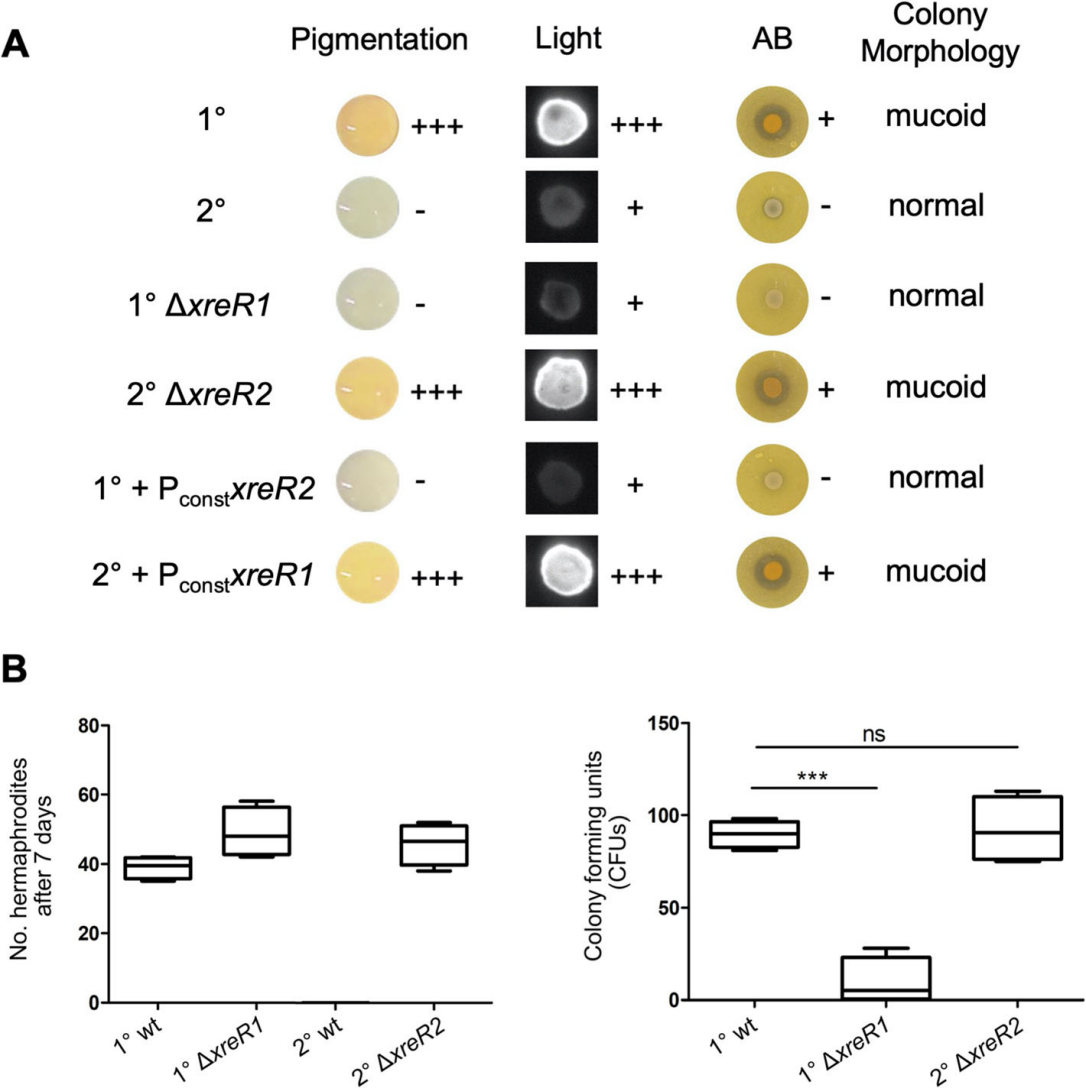
Phenotypic differences in *P. luminescens* 1° and 2° cells as well as *xreR1* and *xreR2* mutant and overproduction strains. **a** Pigmentation, bioluminescence, antibiotic (AB) production and mucoid colony forming of *P. luminescens* 1° and 2° wildtype, *P. luminescens* 1° Δ*xreR1* and *P. luminescens* 2° Δ*xreR2* mutant strains, as well as *P. luminescens* strains that carry chromosomally integrated extra copies of *xreR1* in 2° cells (2° + P_const_*xreR1*) and of *xreR2* in 1° cells (1° + P_const_*xreR2*), respectively, each under the control of the constitutive promoter P_*tac*_ overproducing the XRE-regulator of the respective other cell variant. **b** Nematode symbiosis bioassays. Number of *H. bacteriophora* hermaphrodites developed on *P. luminescens* 1° and 2° wildtype (wt) as well as *P. luminescens* 1° Δ*xreR1* and *P. luminescens* 2° Δ*xreR2* mutant strains after 7 days (left panel), and the number of the respective *P. luminescens* cells isolated from the infective juveniles (IJ) given in colony forming units (CFUs) per IJ after 21 days (right panel). The asterisks (***) indicate statistically significant differences with a *p*-value smaller than 0.001. ns = not significant. Error bars represent standard error of three independently performed experiments

To further analyze the effect of both regulatory genes on the 1° and 2° specific phenotypes, we deleted *xreR1* and *xreR2* in the respective cell form. As *xreR2* was higher expressed in 2° cells we generated 2° cells lacking this gene (2° Δ*xreR2*) as well as 1° cells lacking *xreR1* (1° Δ*xreR1*) since this gene was higher expressed in 1° cells. Then, we analysed the most distinct *P. luminescens* phenotypes to distinguish 1° and 2° cells from each other, which is pigmentation, bioluminescence antibiotic production, and mucoid colony morphology (Fig. 2a). For both 1° Δ*xreR1* and 2° Δ*xreR2* mutant we observed a drastic reversion in the distinct 1° and 2° phenotypes, respectively (Fig. 2a). In contrast to wildtype 2° cells, a red pigmentation of 2° Δ*xreR2* cells could be observed, which usually is a 1°-specific feature. Vice versa, we observed a loss of pigmentation in the 1° Δ*xreR1* strain. The same was found for the 1°-specific features bioluminescence and antibiotic production, which were present in 2° Δ*xreR2* cells. Additionally, 2° Δ*xreR2* cells formed mucoid colonies on agar-plates, which is also a 1°-specific feature. In contrast, also disruption of the *xreR1* gene from 1° cells lead to a reversion to the 2° phenotype. The 1° Δ*xreR1* strain was not bioluminescent anymore and did not produce antibiotics. Furthermore, the 1° Δ*xreR1* cells formed non-mucoid colonies and thereby exhibited the 2°-specific phenotype (Fig. 2a). In summary, both deletion strains exhibit the phenotype of the respective other cell form, in the most predominant phenotypes of 1° and 2° cells (Table 1).

**Table 1:** Phenotypes caused by altering the XreR1 or XreR2 number in *P. luminescens* 1° and 2° cells. The *P. luminescens* strains 2° Δ*xreR2*, 2° + P_const_*xreR1* and 1°Δ*xreR1* + P_const_*xreR1* exhibited the 1° phenotype while the *P. luminescens* strains 1° Δ*xreR1*, 1° + P_const_*xreR2* and 2° Δ*xreR2* + P_const_*xreR2* showed the 2° phenotype

1° phenotype	2° phenotype
1° wildtype	2° wildtype
2° Δ*xreR2*	1° Δ*xreR1*
2° + P_const_*xreR1*	1° + P_const_*xreR2*
1° Δ*xreR1* + P_const_*xreR1*	2° Δ*xreR2* + P_const_*xreR2*

Then, we investigated the effects of increased *xreR2* or *xreR1* levels in the respective other cell variant. Therefore, we chromosomally integrated extra copies of *xreR1* into 2° cells (2° + P_const_*xreR1*) and of *xreR2* into 1° cells (1° + P_const_*xreR2*), respectively, each under the control of the constitutive promoter P_*tac*_. In both 1° and 2° cells, the over-production of the respective other XRE-regulator resulted in phenotypic switching to the other cell variant regarding pigmentation, bioluminescence, antibiotic synthesis and colony morphology (Fig. 2a). The induced phenotypic switch in the deletion strains could successfully be reversed by chromosomally inserting extra copies of the respective gene (data not shown) leading to three strains per phenotype, which were generated by solely altering *xreR2* or *xreR1* levels (Table 1).

Finally, we investigated the effect of *xreR1* and *xreR2* gene deletions on nematode symbiosis. For that purpose, *P. luminescens* 1° and 2° cells as well as *P. luminescens* 1° Δ*xreR1* and *P. luminescens* 2° Δ*xreR2* were exposed to axenic *Heterorhabditis bacteriophora* nematodes, the native symbiosis partner of *P. luminescens*. As it can be seen in Fig. 2b, deletion of *xreR1* did not lead to a loss of nematode growth and development as the number of hermaphrodites grown on *P. luminescens* 1° Δ*xreR1* after 7 days were comparable to 1°. However, *P. luminescens* 1° Δ*xreR1* cells showed a huge decrease in the ability to colonize their symbiosis partners since the number of bacterial cells isolated from infective juveniles (IJs) after 30 days was decreased up to 90%. In contrast, deletion of *xreR2* in 2° cells fully restored nematode growth and development as well as the possibility to colonize the IJs to a 1° wildtype level (Fig. 2b). This showed that XreR1 is only partly essential for the 1° phenotype regarding nematode symbiosis, whereas XreR2 is essential for both nematode development and colonization loss of the 2° phenotype.

### Structural properties of XreR1 and XreR2

**Fig. 3:**
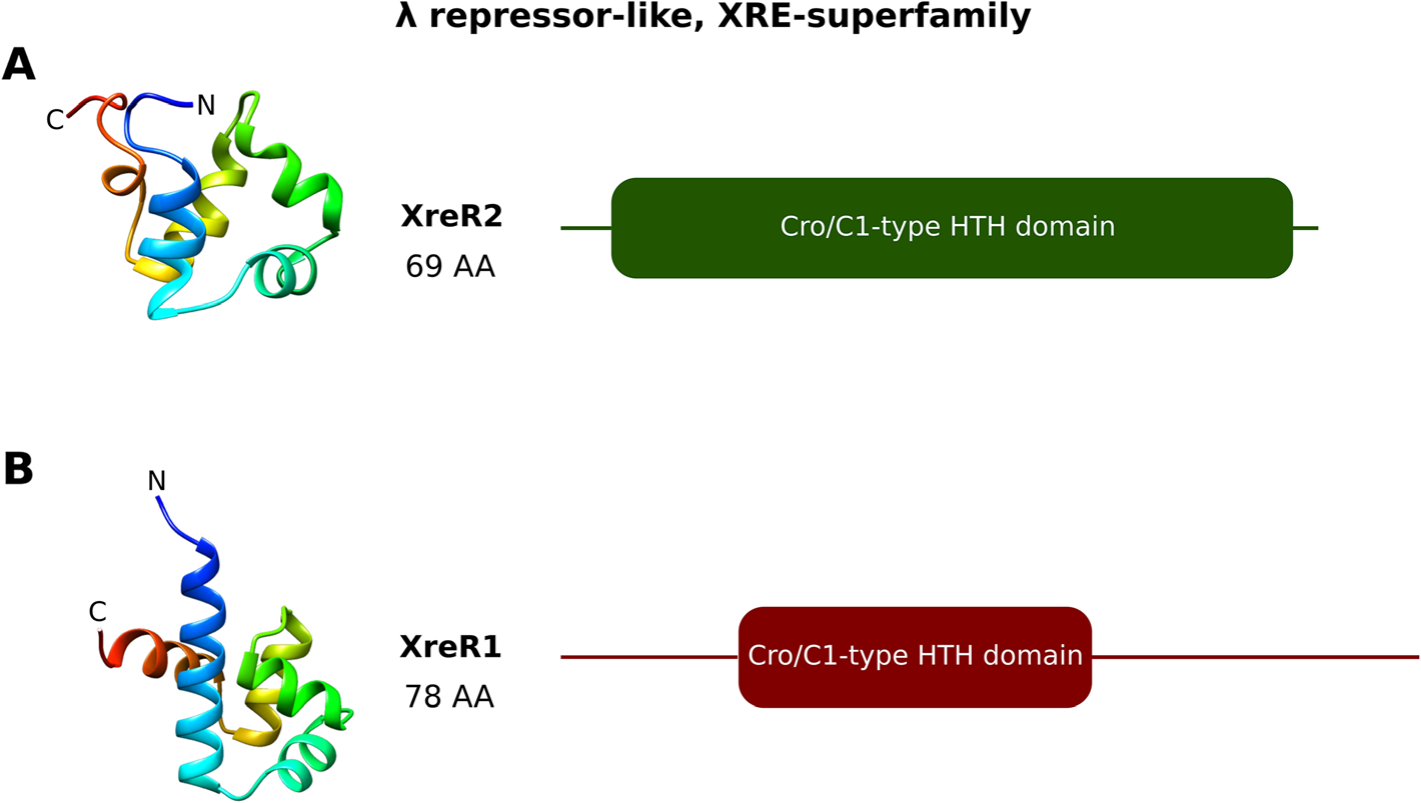
Structure and domain prediction of XreR2 and XreR1. **A** The 69 amino acids (AA) long XreR2 protein is predicted to comprise 5 helices and contains a lambda repressor-like (Cro/C1) HTH DNA-binding domain ranging from position 2 to 68 (**a**). XreR1 comprising 78 AA is predicted to belong to the same superfamily and also forms 5 helices with a Cro/C1 HTH domain reaching from AA 12 to 69 (**b**)

To get more insights about the function of XreR1 and XreR2 the amino acid composition of both were analyzed using Phyre2 [14]. It turned out that both *xreR1* and *xreR2* encode lambda (λ) repressor-like proteins of the same superfamily, the XRE-transcriptional regulators. For XreR2 the highest homology was found to the DNA-binding protein Ner of the *Enterobacteria* phage Mu with the fold library ID d1nera_1. With coverage of 97%, 100% confidence and 59% sequence identity a structure XreR2 was predicted. According to this model it consists of five α-helices and no β-strands. Domain predictions revealed that the 69 amino acid long transcription factor solely consists of a lambda repressor like helix-turn-helix (HTH), also called Cro/C1 HTH DNA-binding domain. A signaling domain could not be identified (Fig. 3a).

The structure prediction for the slightly bigger 78 amino acid long protein XreR1 revealed a highly similar pattern. It also consists five α-helices and is predicted to only harbor a DNA-binding domain. With a coverage of 97%, 99.7% confidence and 42% sequence identity XreR1 was identified to belong to the SinR domain-like family (fold library ID: d2b5aa1) which, according to prediction, also exclusively harbors a Cro/C1-type HTH-domain (Fig. 3b).

### DNA binding of XreR1 and XreR2

Alteration of both, *xreR1* and *xreR2* levels either in *P. luminescens* 1° or 2° cells induced the opposite phenotype in the respective cell type. As both transcriptional regulators exclusively harbor an HTH DNA-binding domain, we attempted to identify direct DNA targets. Here, we started with promoter regions of two of the most predominant 1°-specific traits: P_*luxC*_, the promoter of the *lux* operon that is responsible for bioluminescence and P_*antA*_, the promoter of the *ant* operon responsible for AQ production and therefore pigmentation [4]. Additionally, we analyzed binding of XreR1 and XreR2 to both P_*xreR1*_ as well as P_*xreR2*_ to investigate putative auto-regulatory functions as well as putative effects of one protein onto the expression and therefore synthesis of the respective other protein. Finally, we examined interaction of XreR1 or XreR2 with the promoter region of the operon *PluDJC_21245/50* (P_*pTAS*_), which is genetically clustered with the two regulatory genes since *xreR2* is located directly up- and *xreR1* closely downstream of the *pTAS* operon. With 70% or 64% of identity of PluDJC_21245 and PluDJC_21250 to CcdA and CcdB, respectively, of the toxin/antitoxin system (TAS) in *Escherichia coli* the two corresponding genes in *P. luminescens* were thereby termed *ccdA*-like (*PluDJC_21245,* putative antitoxin) and *ccdB*-like (*PluDJC_21250,* putative toxin). However, CcdB-like seems to be truncated resulting in only 71% coverage of *E. coli* CcdB. In general, TAS are known to be involved in persister cell formation of different bacterial species another kind of phase variation [15]. Since both genes of this putative TAS (pTAS) CcdAB-like are also known to be up-regulated in 2° compared to 1° cells [7] and because of its close proximity to *xreR2* as well as *xreR1* pTAS might play a role in the phenotypic switching process of *P. luminescens*.

**Fig. 4:**
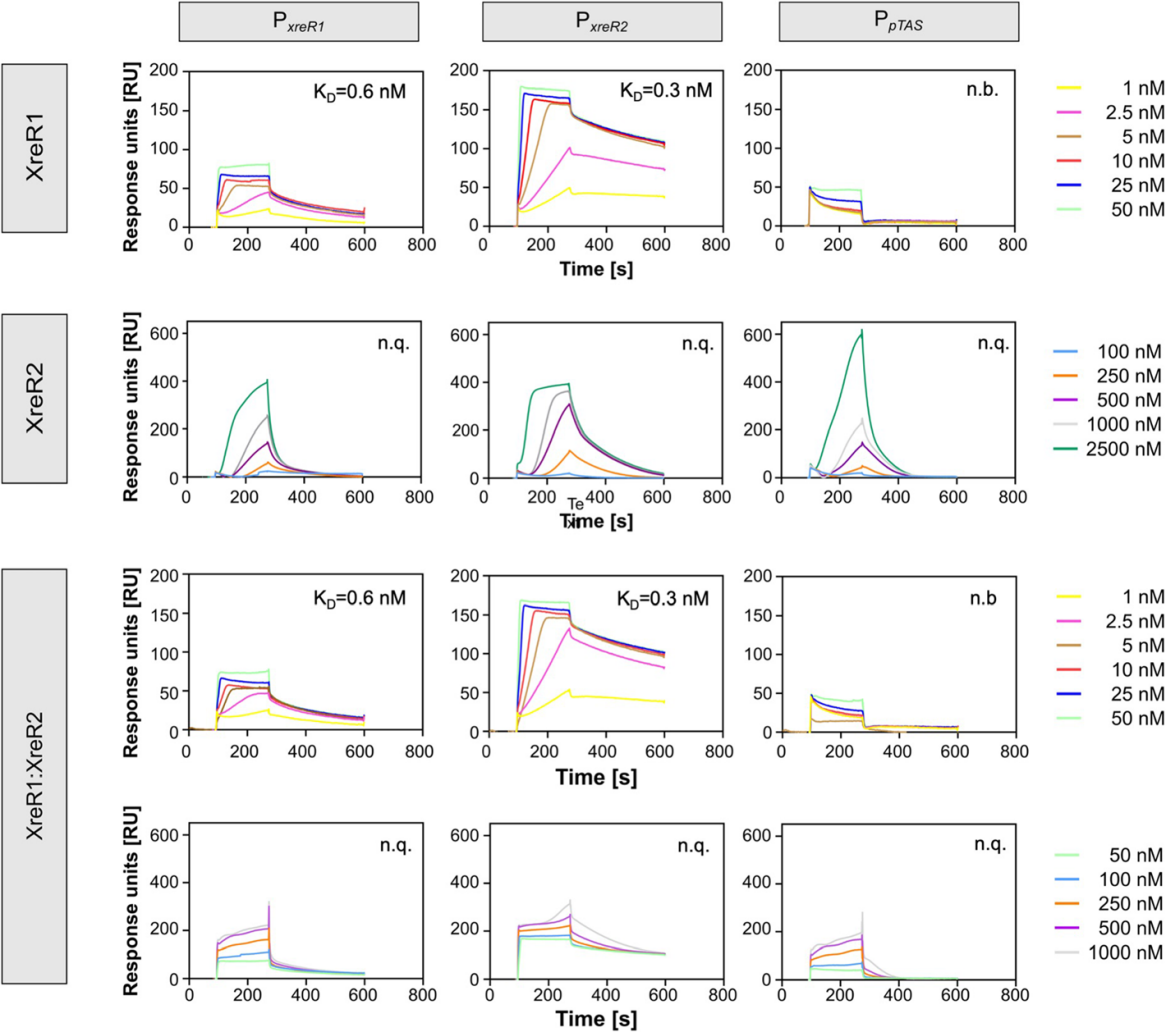
Binding kinetics of XreR2 and XreR1 to different promoter regions. Binding kinetics were determined using SPR spectroscopy**.** The three promoters P_*xreR2*_, P_*xreR1*_ and P_*pTAS*_ were immobilized onto a SA sensor chip and various concentrations of XreR1 (1 nM, 2.5 nM, 5 nM, 10 nM, 25 nM, and 50 nM), XreR2 (100 nM, 250 nM, 500 nM, 1000 nM, 2500 nM) or a 50:50 mixture of both XreR1 and XreR2 (1 nM, 2.5 nM, 5 nM, 10 nM, 25 nM, 50 nM, 100 nM, 250 nM, 500 nM, 1000 nM), respectively, were injected. For better comparability to the sensorgrams using solely XreR1 and XreR2, both sensorgrams using lower and higher XreR1:XreR2 concentrations are shown. All sensorgrams represent one characteristic of three independently performed experiments. n.b. = no binding; n.q. = not quantifiable

Initial Microscale Thermophoresis (MST) analysis indicated binding of XreR2 to P_*xreR2*_, P_*pTAS*_ as well as P_*xreR1*_ (data not shown). XreR1 bound to its own promoter P_*xreR1*_ as well as to P_*xreR2*_. However, none of both displayed binding to P_*luxC*_ or P_*antA*_ indicating no direct regulation of bioluminescence or AQ production by XreR1 and XreR2, respectively. We therefore investigated the binding kinetics of XreR1 and XreR2 with the three identified DNA targets P_*xreR1*_, P_xreR2,_ and P_*pTAS*_ using Surface Plasmon Resonance (SPR) spectroscopy (Fig. 4). For that purpose, the respective promoter regions were immobilized onto streptavidin chips using biotin labeled DNA. XreR1 bound with high affinity due to high association and low dissociation rates of the protein from the DNA (K_D_ = 0.6 nM; *k*_a_ = 4.5E+06 1/Ms.; *k*_d_ = 2.9E-3 1/s) of its own promoter, indicating a strong and stable binding of XreR1 to the selected promoter regions. The interaction was slightly stronger to the P_*xreR2*_ promoter displaying a K_D_ value of 0.3 nM (*k*_a_ = 2.8E+05 1/Ms.; *k*_d_ = 8.9E-4 1/s) due to slower dissociation rates. Both interactions seem to be very stable as the determined disassociation rates were very low. No binding of XreR1 to P_*pTAS*_ could be observed. Furthermore, binding of XreR2 to P_*xreR1*_, to its own promoter P_*xreR2*_ as well as to P_*pTAS*_ was observed. However, even upon applying 2500 nM of XreR2 no saturation of the binding could be observed suggesting a highly complex mode of binding events including self-interaction of XreR2 at high concentrations. The shape of the binding curves supports the idea that the protein forms oligomers and is thereby able to bind to the DNA. Consequently, no K_D_ value could be calculated using the 1:1 binding algorithm due to the mix of different binding events. However, the overall binding affinity of XreR2 to the DNA seemed much lower compared to XreR1 as no binding was observed at low concentrations, where XreR1 already reached maximum binding capacity. Since both XreR1 and XreR2 bound to the similar promoter regions, we tested if both proteins could influence DNA-binding of the respective other protein and tested a 50:50 mixture of XreR1 and XreR2 on binding to the three promoter regions. At low concentrations, where XreR1 already bound to P_*xreR1*_ and P_xreR2_, no influence of XreR2 was observed. The sensorgrams were comparable to those of solely XreR1 to P_*xreR1*_ (K_D_ = 0.6 nM; *k*_a_ = 5.7E+06 1/Ms.; *k*_d_ = 3.4E-3 1/s) and P_*xreR2*_ (K_D_ = 0.3 nM; *k*_a_ = 3.5E+06 1/Ms.; *k*_d_ = 1.0E-3 1/s). However, at high concentrations no binding of XreR2 was detected when injecting the XreR1:XreR2 combination. Binding to all three promoters P_*xreR1*_*,* P_*xreR2*_ and P_*pTAS*_ remained steady state, and was probably due to maximal binding of XreR1. This might be explained with the theory that XreR1 and XreR2 share the same DNA binding site and at high concentrations all binding sites are already occupied by XreR1 so that XreR2 cannot interact with the DNA anymore. Another possibility would be that XreR1 and XreR2 interact at the protein level and that high concentrations of unbound XreR1 outcompete XreR2 due to a higher affinity of XreR1 and XreR2 to each other compared to the XreR2-DNA affinity.

### The XreR1/XreR2 regulation network

**Fig. 5:**
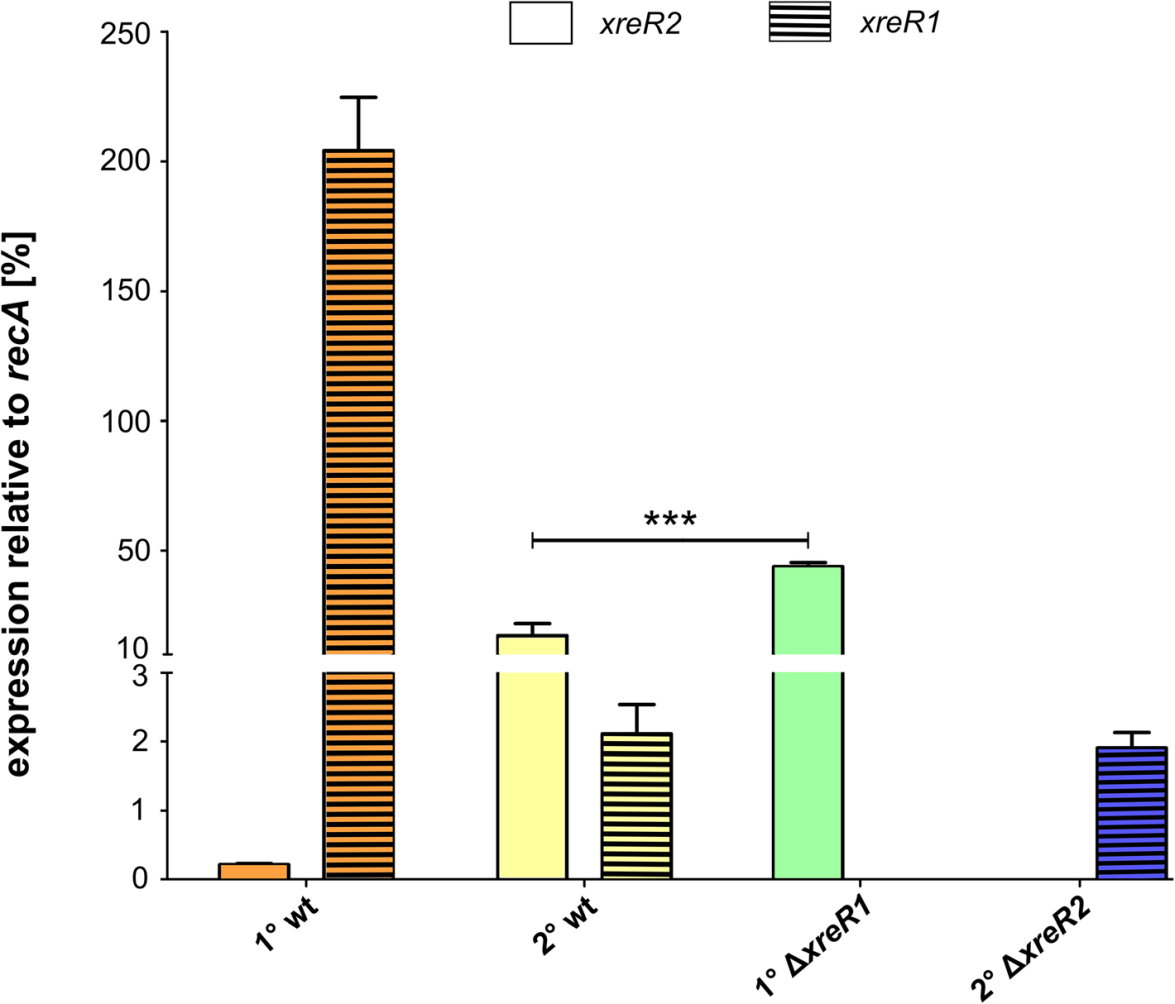
Relative expression levels of *xreR2* and *xreR1* in wildtype and XRE-deletion strains. To compare gene expression of *xreR2* (blank bars) and *xreR1* (striped bars) in *P. luminescens* 1° (orange), *P. luminescens* 2° (yellow), *P. luminescens* 1° Δ*xreR1* cells (green) and *P. luminescens* 2°Δ*xreR2* (blue) mRNA was harvested during exponential growth phase and analyzed via qRT-PCR. Expression levels are depicted in percent, relative to expression of the housekeeping gene *recA*. The asterisks (***) indicate statistically significant differences with a *p*-value smaller than 0.001. Error bars represent the standard deviation of three independently performed experiments. wt = wildtype

SPR analysis revealed a high affinity of XreR1 to bind P_*xreR2*_ as well as binding with lower affinity between XreR2 and P_*xreR1*_. Consequently, we investigated the effects of these binding events on the expression of the respective target genes and quantified expression of *xreR1* and *xreR2* in the 1° Δ*xreR1* and 2° Δ*xreR2* cells, respectively, using qRT-PCR (Fig. 5). Here, *xreR2* seems to be negatively controlled by XreR1 as *xreR2* levels increased in the Δ*xreR1* strain. On the other hand, no significant difference of *xreR1* expression between 2° wildtype and the 2° Δ*xreR2* strain could be observed. Thus, although 2° Δ*xreR2* cells display the 1° phenotype, *xreR1* levels of 1° wildtype were not restored here. This leads to the assumption that *xreR1* expression is not under control of XreR2 and that solely the absence of *xreR2* is sufficient to induce the 2° phenotype.

**Fig. 6:**
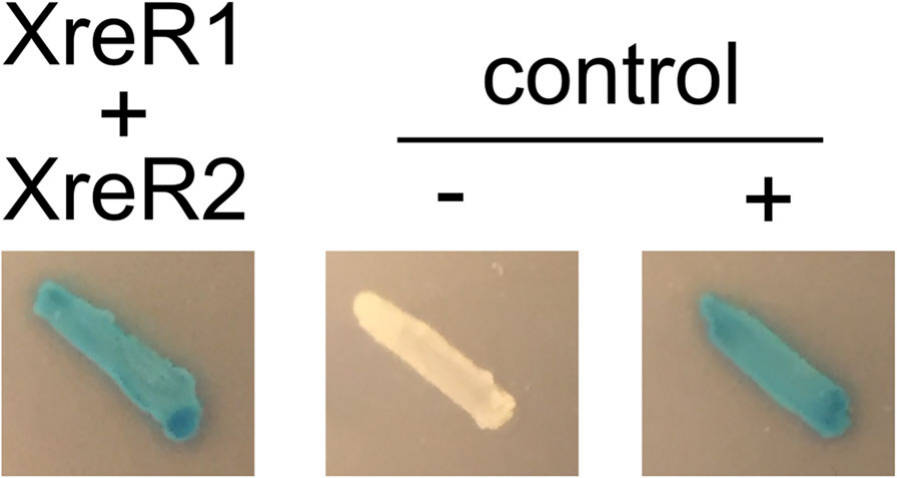
Interaction between XreR1 and XreR2. Bacterial two-hybrid assays to analyze putative binding between XreR1 and XreR2. *E. coli* BTH101 was co-transformed with pUT18-*xreR2* and pKT25-*xreR1*, and then plated on LB agar plates containing X-Gal and IPTG. The empty vectors pUT18 and pKT25 as well as pUT18-zip and pKT25-zip served as negative (−) and positive control (+), respectively. Blue color of the respective reporter strain indicates protein-protein interaction

In addition to protein-DNA interaction we also analyzed a putative interaction between both proteins, XreR1 and XreR2. Therefore, we performed bacterial two hybrid assays (Fig. 6). And indeed, blue colored colonies of the *E. coli* BTH101 cells harboring both plasmids (pUT18-*xreR2* and pKT25-*xreR1*) indicated interaction of XreR1 and XreR2, or vice versa, suggesting a direct regulation of activity by interplay between the two regulators.

### Effect of the toxin/antitoxin (TAS) components CcdA-like CcdB-like on the growth of *P. luminescens*

**Fig. 7:**
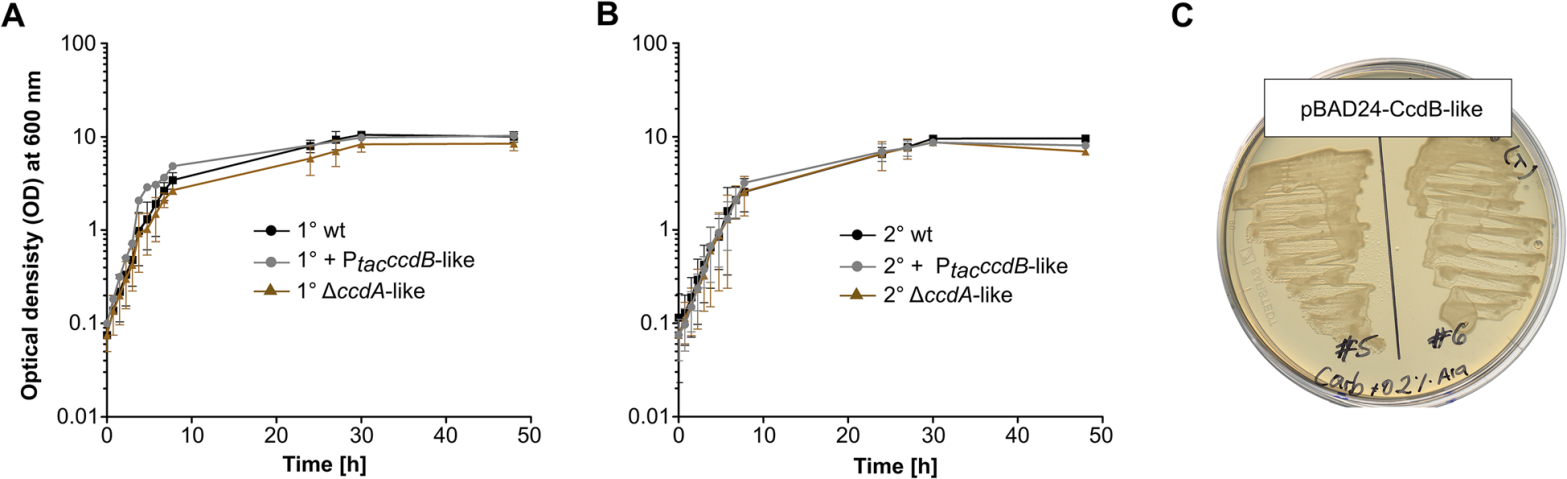
Effect of the pTAS components CcdA-like CcdB-like on the growth of *P. luminescens* 1° and 2° cells*.* To analyze whether CcdB-like also acts as a toxin was overexpressed *ccdB*-like in *P. luminescens* 1° as well as 2° cells. Furthermore, we deleted the cognate putative anit-toxin encoding gene *ccdA*-like in both cell forms and measured growth. Putative effects on the bacterias’ fitness were analyzed by measuring growth over time comparing 1° wildtype (1° wt) to the toxin overexpressing strain (1° + P_*tac*_*ccdB*-like) and the strain lacking the antitoxin 1° Δ*ccdA*-like (**a**) as well as 2° wildtype (2° wt) to the toxin overexpressing strain (2° + P_*tac*_*ccdB*-like) and the strain lacking the antitoxin 2° Δ*ccdA*-like (**b**). Additionally, *ccdB*-like was overexpressed in *E. coli* cells and growth was monitored growth on agar plates (**c**)

The SPR analysis indicated a strong binding of XreR2 to P_*pTAS*_. The pTAS CcdAB-like is similar to the CcdAB TAS of *E. coli* where in absence of the antitoxin (CcdA), the toxin (CcdB) targets the bacterial DNA-gyrase causing cell death by inducing DNA breaks [16]. Therefore, we attempted to investigate if CcdB-like of *P. luminescens* can induce cell death or if it might exhibit a different functionality and thus could have other regulatory tasks e.g. to be involved in the downstream regulation of phenotypic switching in *P. luminescens*. For that purpose, we created knock-in strains overexpressing the *ccdB*-like gene in 1° and 2° cells. Additionally, we generated strains lacking the antitoxin by deleting *ccdA*-like in 1° and 2°, respectively, and analyzed growth of 1° and 2° cells (Fig. 7). Neither toxin overexpressing 1° + P_*tac*_*ccdB*-like cells nor the antitoxin knock-out strain 1° Δ*ccdA*-like exhibited a decrease in fitness as growth was comparable to the 1° and 2° wildtype cells (Fig. 7a). Furthermore, there were also no hints of increased cell death in the 2° + P_*tac*_*ccdB*-like as well as the 2°Δ*ccdA*-like strain as the growth was also comparable to the the 1° and 2° wildtype (Fig. 7b). As the pTAS is similar to the CcdAB system of *E. coli* we overproduced the toxin homolog CcdB-like in *E. coli* Dh5α-λ*pir* cells using the arabinose inducible promoter of pBAD24 vector. Upon arabinose addition we could not observe a disadvantage in growth compared to the non-induced cells on agar-plates (Fig. 7c) as well as in liquid culture (data not shown). In summary, although homologous to the *E. coli* CcdAB TAS, the pTAS of *P. luminescens*, which is under regulatory control of XreR2 does not function as toxin/antitoxin system and might therefore fulfil a different regulatory effect in *P. luminescens*.

## Discussion

The appearance of two distinct phenotypically different cell forms makes *P. luminescens* to a perfect model organism to study bacterial phenotypic heterogeneity. However, the regulation of phenotypic heterogeneity in *P. luminescens* is still not completely understood. Here we identified two novel transcriptional regulators, XreR2 as well as XreR1, to have major impact on phenotypic switching in *P. luminescens*. Both belong to the XRE (xenobiotic response element) superfamily, which is the second most frequently occurring regulator family in bacteria [17]. Proteins of this family are usually activated by interaction with environmental signals ranging from small effector molecules to large proteins [18, 19]. Though, XreR2 and XreR1 were predicted to exclusively harbor a helix-turn-helix (HTH) DNA-binding domain similar to the Cro/C1 repressor protein of *λ* phage, comprising five α-helices without any additional domain. In XRE-regulators this Cro/C1-HTH domain, always located N-terminally, [17, 20, 21] is highly conserved, while the C-terminal regulatory domain is variable [22]. However, the XRE subfamily of *λ*-like repressors is one of the best examples for simplest architectures as they almost entirely consist of a standalone HTH [23]. Several structures of Cro/C1-type transcriptional regulators have been resolved in the past. Here, similar as for XreR2 and XreR1 the DNA-binding domain consists of five α-helices which are highly conserved inside but much less at the extremities. Usually, the HTH motif which binds the DNA comprises the 2nd and 3rd helices. The remaining ones are involved in DNA-contacts and are referred to as recognition helices [24].

We demonstrated that XreR2 binds to its own promoter. As expression of *xreR2* is essential to maintain the 2° phenotype of *P. luminescens*, it seems likely that XreR2 positively auto-regulates its own expression, also taking into account that the regulator is about 500-fold up-regulated in 2° compared to 1° cells [7]. Furthermore, we could show that XreR2 binds to P_*pTAS*_, the promoter of the genes encoding the putative TAS system CcdAB-like (PluDJC_21245/50). Since both of these genes are also higher expressed in 2° than in 1° cells [7] the *pTAS* expression is also presumably activated by XreR2. Lastly, XreR1 also binds to its own promoter again suggesting a positive feedback loop.

Transcriptional regulators with phage-like HTH domains have usually repressing functions. However, in *Corynebacterium glutamicum* a member of the XRE family, ClgR, activates an operon encoding Clp proteases which then in turn recognize and degrade defective proteins [25]. Furthermore, recently an XRE transcriptional regulator of *Streptococcus suis,* SrtR, was found to be enhance the cells tolerance towards oxidative stress and high temperature [26].

Binding kinetics of XreR2 via SPR did not go into saturation for none of the tested promoters indicating no 1:1 binding of XreR2. Phage repressor-like proteins of the XRE superfamily are one example of proteins with the simplest HTH architecture. Almost every member of this family is built up by a standalone HTH. Among them some proteins harbor short extensions that are used to support protein folding and DNA contact [27]. Therefore, the initiate binding of XreR2 to its DNA targets could allow the protein to fold properly and so enables binding the specific site.

XreR2 showed high homology to the Ner regulator of the *Enterobacteria* phage Mu. A Ner-like regulator has already been demonstrated to be involved in phenotypic switching of *Photorhabdus temperata* K122 [28]. Overproduction of the *ner*-gene in 1° cells resulted in repression of 1°-specific features. However, inactivation of *ner* gene in 2° cells was not sufficient to revert to 2° cells to the 1° phenotype. This showed that Ner cannot be solely responsible for regulating the 2° phenotype, so it might be possible that XreR2 interacts and/or oligomerizes with Ner to downstream regulate selected 2°-specific features.

**Fig. 8:**
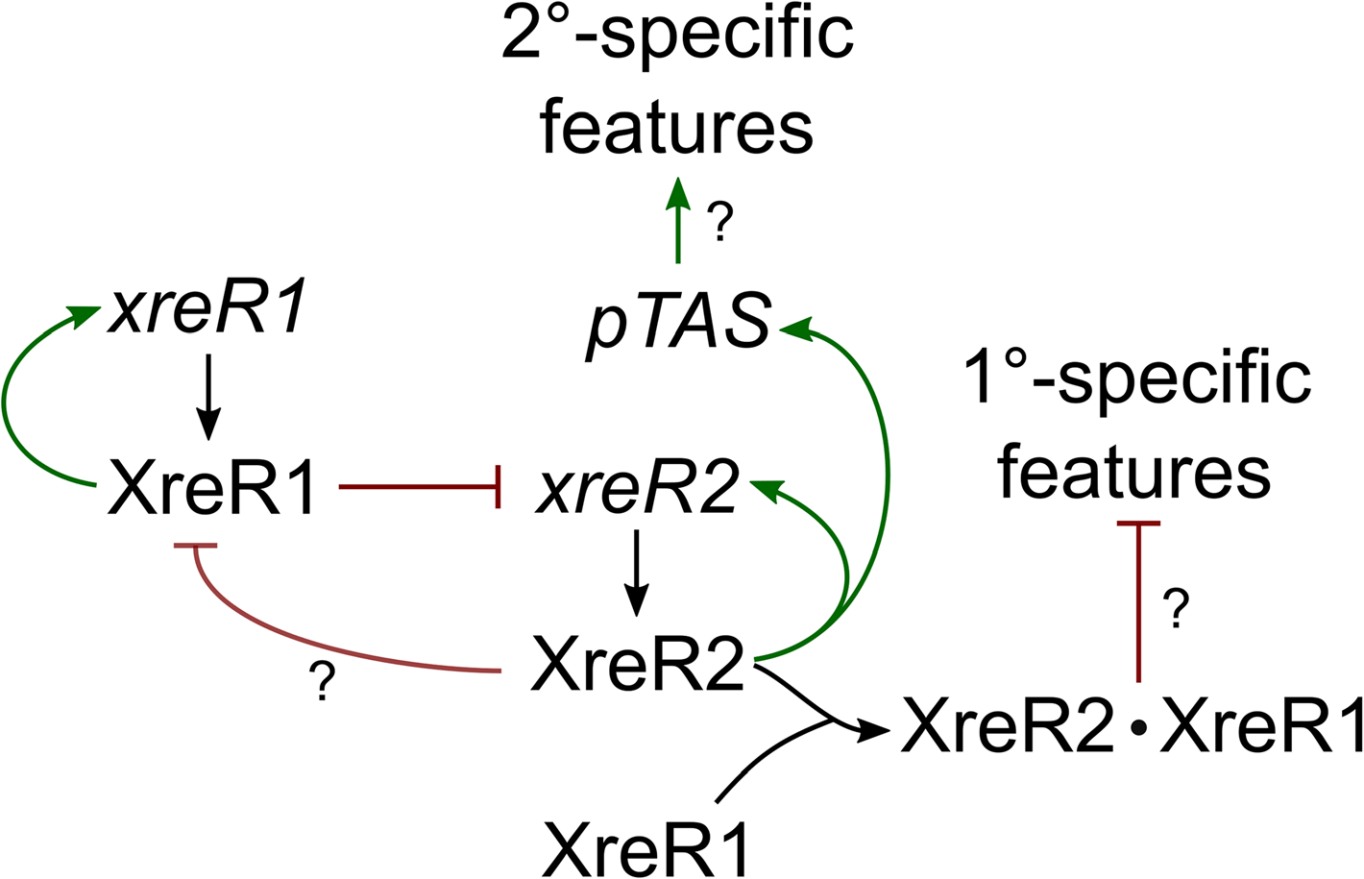
Model of gene regulation via XreR1 and XreR2. XreR1 binds to its own promoter, most probably leading to a positive feedback loop. Furthermore, it represses the expression of *xreR2* and thereby maintains the 1° phenotype. XreR2 in turn binds to XreR1 thereby putatively re-enforcing its own expression. The XreR1-XreR2 complex might directly represses 1°-specific features inducing the 2° phenotype. Additionally, XreR2 most probably activates expression of the TAS-derived *ccdAB*-like system which could maintain the 2° phenotype by activating 2°-specific features. Green: activation; red: inhibition

One of the best-studied XRE transcriptional regulators with a DNA-binding domain similar to that of the phage repressor proteins, C1 and Cro, is SinR of *Bacillus subtilis* [29], which represses biofilm formation by binding to the respective *eps* promoter. It has been shown that SinR represses the expression of *slrR* that encodes another XRE-family member, SlrR, which in turn represses SinR via direct binding. Thus, SinR and SlrR create a double negative feedback loop directly controlling genes involved in cell separation and motility. Upon activation of that loop the cell becomes time-dependently locked in a high SlrR state [30]. The binding of XreR1 to P_*xreR2*_ and the increase of *xreR2* levels in the 1°Δ*xreR1* strain also indicate a repression of *xreR2* by XreR1. Furthermore, both proteins seem to interact with each other. Therefore, XreR1 and XreR2 might also constitute an epigenetic switch comparable to the one of SinR and SlrR of *B. subtilis* (Fig. 8). No binding of XreR2 or XreR1 to the P_*luxC*_ or P_*antA*_ promoter could be detected indicating no direct repression of bioluminescence or AQ-production. Here, it is worth mentioning that interaction assays were performed using either only XreR1 or XreR2. In the SinR/SlrR model of *B. subtilis* the respective genes are regulated by a complex of both proteins [30]. Therefore, a mixture of both, XreR1 and XreR2, might be needed to enable binding to promoter regions of 1°- and 2°-specific phenotypes and thereby repressing or activating gene expression (Fig. 8). In *B. subtilis* the antagonist of SinR, SinI, gets activated during stationary phase and binds to SinR thereby releasing P_*eps*_ and promoting biofilm formation [18, 31]. This suggests that the phenotypic switch is also reversible in *P. luminescens* DJC. However, the respective signal to trigger that conversion is still unknown and a switch from the 2° to the 1° phenotype has, to our knowledge, not yet been reported for *P. luminescens*.

Other regulators have been identified before to be mainly involved in the regulation of phenotypic heterogeneity in *P. luminescens*, which is HexA and Hfq. The LysR-type transcriptional regulator HexA acts as a versatile repressor of 1°-specific traits [6, 12]. Deletion of *hexA* in 2° cells restored the 1° phenotype, while extra copies of *hexA* in 1° cells were sufficient to induce the 2° phenotype [12]. This is comparable to the gene deletion and overproduction effects of *xreR2* and contrary to those of *xreR1*. Therefore, it was likely that HexA might somehow interfere with the XreR1/XreR2 regulation network. Expression of *hexA* is under control of the Hfq RNA chaperone at the post-transcriptional level [32]. The Hfq-regulated small-RNA ArcZ base-pairs with the HexA-mRNA to repress synthesis of HexA and thereby of the HexA downstream-regulated genes [33]. However, unlike *hexA*, *xreR2* is not repressed by the RNA-chaperone Hfq since mRNA levels of *xreR2* were not altered in a Δ*hfq* strain (Nick Tobias, Helge Bode, Goethe-Universität Frankfurt, personal communication), while *hexA* expression is about 60-fold increased [32]. Furthermore, the up-regulation of *hexA* in the Δ*hfq* strain did neither lead to an activation of *xreR2* nor to a repression of *xreR1* expression. Additionally, expression levels of both *xreR2* and *xreR1* were not altered in a D*hexA* strain (Nick Tobias, Helge Bode, personal communication). Furthermore, *hexA* is also not regulated downstream of XreR1 or XreR2 since we could not detect a direct interaction of both regulators to the P_*hexA*_ promoter using SPR (J.B., M.L. and R.H., unpublished). However, an interaction between HexA and XreR1/XreR2 cannot be completely ruled out, because although *hexA* is higher expressed in the Δ*hfq* strain, it could be inactive due to the lack of a putative signal sensed by HexA. Furthermore, HexA could directly interact with either XreR1/XreR2 at the protein level to influence activity the XRE regulators or the other way round.

Recently, we demonstrated that *P. luminescens* 2° cells specifically interact with plant roots, uncovering a different life cycle for the bacteria in soil in absence of the nematode partners [9]. Transcriptome comparison of 2° cells in presence and absence of plant root exudates highlighted another two XRE-like regulators (PluDJC_03960 and PluDJC_10030), which were highly upregulated in 2° cells upon exposition towards plant root exudates [9]. This suggested that XRE-like regulators are not only involved in phenotypic switching from 1° to 2°, but also in the adaptation of 2° cells to plants. Therefore, it is conceivable that the regulation of heterogeneity by XRE-like regulators and a permanent establishment of a specific phenotype or adaptation towards the specific eukaryotic host is more complex, and not only based on the interplay between XreR1 and XreR2.

Lastly, the role of the putative TAS CcdAB-like still remains elusive. There are several TAS which are described to be involved in persistence. Persister cell formation is one of the best-studied bacterial phenotypic heterogeneity formed using the bet-hedging strategy. Hereby, upon antibiotic treatment, single cells reversibly switch into a transient growth arrested state, which allows them to survive the stress situation [34]. The CcdB protein of *E. coli* owes its toxicity to the last three C-terminal amino acid residues tryptophan 99, glycine 100 and isoleucine [35]. Sequence analysis revealed that CcdB-like of *P. luminescens* is C-terminally truncated including the respective amino acids and thereby lost the amino acid residues responsible for CcdB toxicity in *E. coli*. This could explain the absence of an obvious phenotype in the *P. luminescens* Δ*ccdA-*like or P_*tac*_*ccdB*-like strains indicating that the CcdAB-like system arose from a TAS but owns a new function. The idea that the pTAS is involved in downstream regulation of phenotypic heterogeneity in *P. luminescens* is supported by the fact that the corresponding genes are located in close proximity to *xreR2* and *xreR1* and that XreR2 directly binds to the promoter region of the *pTAS* genes. As *ccdA*-like as well as *ccdB*-like were found to be up-regulated in 2° cells [7] a positive regulation of it via XreR2 seems likely. The CcdAB-like system might support to maintain the 2° phenotype by e.g. activating 2°-specific features (Fig. 8). However, the exact mechanism of how pTAS is involved in regulation of phenotypic heterogeneity in *P. luminescens* remains to be elusive.

## Conclusions

We identified two novel XRE-transcriptional regulators, XreR1 and XreR2, which play a major role in the process of phenotypic switching in *P. luminescens.* Both proteins interact with each other and regulate gene expression by interacting with the promoter regions of the target genes including their own and thereby display a complex regulatory network putatively including a double negative feedback loop. However, whether phenotype specific features are directly regulated via a XreR1/XreR2 complex or by other proteins that are under the control of XreR1 or XreR2 has to be elucidated. Finally, the XRE-regulation network might be much more complex and not only based on XreR1 and XreR2, but other XRE-like regulators might come into play not only for switching but also for adaptation to the different eukaryotic hosts of *P. luminescens*.

## Methods

### Bacterial strains and growth conditions

*E. coli* strains MG1655 [36] and DH5αλpir [37] were routinely grown at 37 °C in LB medium [1% (w/v) NaCl; 1% (w/v) tryptone; 0.5% (w/v) yeast extract]. If necessary, 50 μg/ml antibiotic was added into the medium. All *P. luminescens* DJC [2] strains were cultivated aerobically in either LB medium or CASO medium [0.5% (w/v) NaCl, 0.5% (w/v) peptone from soy; 1.5% (w/v) tryptone] at 30 °C. If necessary, the growth medium was supplemented with 50 μg/ml rifampicin (Sigma Aldrich, Deisenhofen). For preparation of agar plates, 1.5% (w/v) agar was added to the respective medium.

### RNA preparation

RNA extraction from *P. luminescens* cultures was performed as described previously [7]. Briefly, three independent cultures of *P. luminescens* DJC 1° or DJC 2° cells were grown to optical densities at 600 nm (OD_600_) of 3 (mid-exponential growth phase) and 10 (early stationary growth phase) and then the total RNA was extracted. For that purpose, the pellets of harvested cells were resuspended in 500 μl ice-cold AE-buffer [20 mM NaAc pH 5.2, 1 mM EDTA pH 8.0]. Then, 500 μl Roti®-Aqua-P/C/I (Roth) and 25 μl 10% (w/v) SDS was added. After mixing the samples were incubated for 30 min at 60 °C under shaking. Subsequently, the samples were placed into the fridge for one night. On the next day the samples were centrifuged with 16.100 rcf for 40 min at 0 °C. Afterwards the supernatant was transferred into 5PRIME Phase Lock Gel Tubes (Quantabio, Beverly, USA), supplemented with 500 μl P/C/I and 50 μl 3 M NaAc pH 5.2 and after mixing the tubes were centrifuged with 16.100 rcf for 10 min at 0 °C. Then the supernatants were mixed with 1 ml 96% (v/v) EtOH and put on − 80 °C for overnight precipitation. Then, the samples were again centrifuged with 16.100 rcf for 30 min at 0 °C and the supernatant was discarded. To wash the pellet, 1 ml 80% (v/v) EtOH was added and subsequently removed by centrifugation with 16.100 rcf for 10 min at 0 °C. This washing step was repeated 2 times. Then, the pellet was air dried for 60 min with open lid and resolved in 100 μl DEPC-treated water. 5 μg of RNA were then treated with DNaseI to remove genomic DNA.

### qRT-PCR

Quantitative reverse transcription-PCR (qRT-PCR) was carried out with three independent total RNA preparations, in each case in triplicates. cDNA was synthesized during the run using Luna® Universal One-Step RT-qPCR Kit (New England Biolabs, Frankfurt, Germany). For that purpose, the reactions were performed according to the protocol provided by the manufacturer. Reactions and melting curves were monitored in the LightCycler (BioRad, München, Germany). Differences in gene expression levels were calculated using the Pfaffl-Method [38] with *recA* serving as housekeeping gene. All data are presented as a ratio of three independent biological replicates. Values are means, ± the standard deviation.

### Generation of plasmids

To generate pNPTS-FAB-ΔxreR2 500 bp upstream (FA) and downstream (FB) of genomic *xreR2* were amplified by PCR using the primer pairs BamHI-xreR2-FA fwd + xreR2-FA-ovl-FB rev and xreR2-FB-ovl-FA fwd + xreR2-FB-EagI rev introducing a BamHI and a EagI restriction site to the 5′ end of the upstream fragment and the 3′ end of the downstream fragment, respectively. Overlap extension PCR was used to fuse the two PCR products which were then cloned into the pNPTs138-R6KT backbone using the BamHI and EagI restriction sites. Correctness of the plasmid was confirmed by PCR using primers check-pNPTS fwd and check-pNPTS rev. Plasmid pNPTS-FAB-ΔxreR1 was generated the same way, however, with different restriction sites. Here EcoRI and EagI were used. Therefore, the respective primer pairs were EcoRI-xreR1-FA fwd + xreR1-FA-ovl-FB rev and xreR1-FB-ovl-FA fwd + xreR1-FB-EagI rev. For pPINT-Ptac-xreR2 and pPINT-Ptac-xreR1 generation a *lacI*-P_*tac*_ fragment (PstI-lacI_Ptac fwd: + Ptac-ovl-blank rev) was fused to either genomic *xreR2* (xreR2-ovl-Ptac fwd: + xreR2-EagI rev) or genomic *xreR1* (xreR1-ovl-Ptac fwd + xreR1-EagI rev) via overlap PCR, respectively, resulting in P_*tac*_-*xreR2* and P_*tac*_-*xreR1* each harboring a 3′-PstI and 5′-EagI restriction site. Afterwards the single fragments were cloned into the empty pPINT backbone. Correctness of the plasmids were checked by sequencing using the primers check-pPINT fwd and check-pPINT rev. To generate pPNPTS-FAB-ΔccdA-like the up- and downstream flanking regions of genomic *ccdA-like* were amplified using the primer pairs BamHI-FA ccdA-like fwd + FA ovl FB ccdA-like rev and FB ovl FA ccdA-like fwd + FB ccdA-like-EagI rev. The resulting amplicons were then fused via overlap extension PCR and thereby FAB harboring a 5′-BamHI and 3′-EagI restriction site was generated. Using the respective restriction enzymes FAB was cloned into the empty pNPTs138-R6KT backbone. Plasmid pNPTS-FAB-ΔccdB-like was achieved by the same procedure. FA was amplified using the primer pair BamHI-FA ccdB-like fwd + FA ovl FB ccdB-like rev and FB was achieved by using primers FB ovl FA ccdB-like fwd and FB ccdB-like-EagI rev. Again, both flanking regions were fused via overlap extension OCR and the resulting FAB fragment was cloned into the pNPTs138-R6KT backbone [39] using the restriction enzyme sites BamHI and EagI. For pPINT-Ptac-ccdB-like generation again the *lacI*-P_*tac*_ fragment was fused to genomic *ccdB*-like amplified with the primers ccdB-like ovl Ptac fwd and ccdB-like-EagI rev via overlap extension PCR. The resulting fragment was then cloned into the empty pPINT vector by utilizing the restriction enzymes PstI and EagI. To gain the plasmid pBAD24-ccdB-like, genomic *ccdB*-like was amplified using the primers NheI-ccdB-like fwd and ccdB-like-XmaI rev. The thereby introduced restriction sites were used to clone the gene into the pBAD24 backbone downstream of the P_*ara*_ promoter. Correctness of all plasmids based on the pNPTs138-R6KT backbone were checked by sequencing using the primers: check-pNPTS fwd and check-pNPTS rev. Integrational plasmids with pPINT backbone were sequenced with the primer pair check pPINT fwd + check-pPINT rev. Correctness of the pBAD24-ccdB-like plasmid was confirmed by sequencing with the following primers: check-pBAD24 fwd + check-pBAD24 rev. For generation of BACTH plasmids pUT18-xreR2 and pKT25-xreR1 the respective genes were amplified from genomic DNA of. *P. luminescens* DJC using primer pairs BamHI_g_xreR2 fwd and xreR2_XmaI rev or BamHI_xreR1 fwd and xreR2_XmaI, respectively, and the corresponding fragments were cloned into pUT18 or pKT25 (Euromedex, Souffelweyersheim) using restriction sites BamHI and XmaI. As positive control for the BACTH assay, plasmids pUT18-zip and pKT25-zip (Euromedex, Souffelweyersheim) were used. All oligo sequences used in this study are listed in Table 2.

**Table 2:** Oligo nucleotides used in this study

Name	Oligo sequence 5′ → 3′
BamHI-xreR2-FA fwd	GCCGGGATCCGTACTTATCAGTTACCACCAACCC
xreR2-FA-ovl-FB rev	CGTCAGTAGATCCATTAATTAATCCTCCGTGTTAC
xreR2-FB-ovl-FA fwd	GATCTACTGACGTAGGTGGCTGTAAATTAAAGTGG
xreR2-FB-EagI rev	ATCCCGGCCGGCGCTTCAACTAAAGGAATAGCC
EcoRI-xreR1-FA fwd	GCCGGAATTCGGTTATCTGAACGATCCTGAAC
xreR1-FA-ovl-FB rev	ATCTGTATCGCGCATTCGATAAGTATCGAACGATG
xreR1-FB-ovl-FA fwd	CGCGATACAGATTAGTGATCTATACCTTATGG
xreR1-FB-EagI rev	ATCCCGGCCG GAAATTGCGCTCGTTACTGCTG
PstI-lacI_Ptac fwd	GCGCTGCAGCATTAATTGCGTTGCGCTCA
Ptac-ovl-blank rev	ATCTGTATCGCGAGAATTCCCTCCTGTGTGAAATTG
xreR2-ovl-Ptac fwd	TCGCGATACAGATATGAAAAATCAAGACTGGCACC
xreR2-EagI rev	ATCCCGGCCGCTACTTTGTGTACCTCGAAGGCC
xreR1-ovl-Ptac fwd	TCGCGATACAGATATGAAAAAACCTAACACGATAAAATC
xreR1-EagI rev	ATCCCGGCCGCTAACTCTTTTTCTCCGCGTC
BamHI-FA 4295 fwd	GCCGGGATCCCTTATCAGACCACGGACTATGAG
FA ovl FB 4295 rev	CGTCAGTAGATCCATAGCACACCTCAGAAACAC
FB ovl FA 4295 fwd	GATCTACTGACGTAAATGCAATTTGTTGTTTATC
FB 4295-EagI rev	ATCCCGGCCGCAACGCTGGGCGCAGGCAAACCTAC).
BamHI-FA 4296 fwd	GCCGGGATCCGCATCATCGACCCTTGCCAATAC
FA ovl FB 4296_rev	CGTCAGTAGATCTTACCAGTTCCTGTTTTCATC
FB ovl FA 4296 fwd	GATCTACTGACGTCAGTTTTCTTCATTTGTGCCGCTCTG
ccdB-like ovl Ptac fwd	TCGCGATACAGATATGCAATTTGTTGTTTATC
ccdB-like-EagI rev	ATCCCGGCCGTTACAGAAAAATATTCATACAG
NheI-ccdB-like fwd	GCTGCTAGCATGCAATTTGTTGTTTATC
ccdB-like-XmaI rev	CAGCCCGGGTTACAGAAAAATATTCATACAG
check-pNPTS fwd	TGCTTCCGGCTCGTATG
check-pNPTS rev	GTAAAACGACGGCCAGTCC
check-pPINT fwd	CCGTTCTGTGCGAATCGTGGAG
check-pPINT rev	GGCCATTGGCACTGATTG
check-pBAD24 fwd	GCCGTCACTGCGTCTTTTACTGG
check-pBAD24 rev	CGCTACGGCGTTTCACTTCTG
Cy5-PxreR2 fwd	[Cy5] CTGATAAGTATCGAATGATGTTAC
PxreR2 rev	GGGTGCCAGTCTTGATTTTTC
Cy5-PxreR1 fwd	[Cy5] CTTAATAAATAATACTTGCGACCAGATTGG
PxreR1 rev	CGTGTTAGGTTTTTTCATTCGATAAG
Cy5-PpTAS fwd	[Cy5] GTGGCTGTAAATTAAAGTGGAGTAG
PpTAS rev	GTTTCATAGCACACCTCAGAAAC
Cy5-PantA fwd	[Cy5] TAATGCAGAAATTATTGCT
PantA rev	CTGAACTATTCCTATCGTTA
Cy5-PpluxC fwd	[Cy5] TTTGTATATAAAGAAGAGCTTG
PluxC	ATTAGCCATCCATTTAATG
Cy5-fryB-300 fwd	[Cy5] GGTGTATCTCATCGTCCATGACAAT
fryB −0 rev	TTGAATTCCGTTAATTCCTCGTTCAG
Btn-PxreR2 fwd	[Btn] CTGATAAGTATCGAATGATGTTAC
Btn-PxreR1 fwd	[Btn] CTTAATAAATAATACTTGCGACCAGATTGG
Btn-PpTAS fwd	[Btn] GTGGCTGTAAATTAAAGTGGAGTAG
BamHI_xreR1 fwd	GCTGGATCCGATGCAATTTGTTGTTTATC
BamHI_xreR2 fwd	CTGGATCCATGAAAAATCAAGACTGGCACCC
xreR1_XmaI rev	CAGCCCGGGTTACAGAAAAATATTCATACAG
xreR2_XmaI rev	CAGCCCGGGCTACTTTGTGTACCTCGAAGGC

### Generation of genomic gene deletions in *P. luminescens*

For deletion of genomic *xreR2* in 2° cells or *xreR1* in 1° cells the plasmids pNPTS-FAB-ΔxreR2 and pNPTS-FAB-ΔxreR1 were used, respectively. The genes were deleted via double homologous recombination as described previously [40]. For that purpose, the respective plasmid was conjugated from *E. coli* S17–1 λ*pir* into 1° or 2° cells and exconjugants were selected as Rif^R^Km^R^ colonies. The pNPTS138-R6KT plasmid contains the *sacB* gene and, after growth in LB broth (with no selection), putative mutants were identified by screening for Rif^R^ Suc^R^ Km^S^ colonies. Successful deletion of *xreR2* or *xreR1* was confirmed by PCR using either the primer pair BamHI-xreR2-FA fwd/xreR2-FB-EagI rev or EcoRI-xreR1-FA fwd/xreR1-FB-EagI, respectively, followed by DNA sequencing.

### Insertion of extra gene copies into *P. luminescens* genome

To chromosomally insert constitutive expressed copies of either *xreR2*, *xreR1* or *ccdB*-like into *P. luminescens* 1° or 2° cells, respectively, the non-coding intergenic region between the two genes *glmS* and *rpmE* was utilized. Therefore, the respective plasmids pPINT-Ptac-xreR2, pPINT-Ptac-xreR1 or pPINT-Ptac-ccdB-like were used. Insertion and backbone depletion were obtained via double homologous recombination as described above. Successful insertion of each gene was checked using again the primers check-pPINT fwd and check-pPINT rev followed by DNA sequencing.

### Phenotypic bioassays

To analyze bioluminescence of *P. luminescens* cultures 1 ml LB were inoculated to an OD_600_ = 1 with overnight cultures of the respective *P. luminescens* variant. Subsequently, 5 μl of the cultures were spotted onto LB plates and incubated at 30 °C. After 48 h bioluminescence was monitored using a Chemiluminescence Imager (Peqlab, Erlangen) using 5 min exposure time. For testing antibiotic activity of *P. luminescens*, soft agar plates supplemented with *Bacillus subtilis* as test strain were used. Briefly, an overnight culture of *B. subtilis* (OD_600_ = 2–3) was added in 1:100 dilution to liquid hand-warm LB agar medium 0.8% (w/v) agar. After the plates were polymerized, 30 μl (OD_600_ = 1.0) of the respective *P. luminescens* DJC strain, was dropped onto the middle of the agar plate and incubated for 48 h at 30 °C. The development of red pigments was visually noted after 3 days of growth of *P. luminescens* DJC 1° and 2° cells on LB plates at 30 °C.

### Symbiosis bioassays

An aliquot of 50 μl of overnight cultures of *P. luminescens* DJC 1° and 2° cells as well as *P. luminescens* 1° Δ*xreR1* and *P. luminescens* Δ*xreR2*, diluted to an OD_600_ of 1.0, were spread in a Z pattern onto the surface of a lipid agar plate [1% (v/v) corn syrup, 0.5% (w/v) yeast extract, 5% (v/v) cod liver oil, 2% (w/v) MgCl_2_·6 H_2_O, 2.5% (w/v) Difco nutrient agar (Becton, Dickinson, Heidelberg, Germany)] using an inoculating loop. The plates were incubated at 30 °C for 3 days before addition of 50 surface-sterilized axenic *Heterorhabditis bacteriophora* TT01 infective juvenile (IJ) nematodes to the bacterial biomass. Nematodes were surface sterilized by washing in a solution 0.4% (w/v) of hyamine (Sigma-Aldrich, Deisenhofen, Germany). The plates were kept at room temperature. Nematode recovery was assessed 7 days after IJ nematodes addition by counting the number of hermaphrodites on the lipid agar plate. The new generations of IJs migrated to the lid of the Petri dish and, after 21 days, these nematodes were collected by washing the lid with PBS buffer to a final volume of 50 ml, and the number of IJs present (i.e the IJ yield) was determined. Colonization levels in the IJs were determined by crushing 50 surface-sterilized IJ nematodes in 100 μl PBS buffer using a hand-held homogenizer and plating the homogenate onto LB agar. Then, the number of colony forming units (CFU) was calculated per single IJ.

### Bacterial adenylate cyclase two-hybrid (BACTH) assays

BACTH assays were performed according to the manufacturer’s instructions (Euromedex, France). In brief, *E. coli* BTH101 cells were co-transformed pUT18-*xreR2* and pKZ25-*xreR1* and plated onto LB plates containing carbenicillin (100 μg/ml), kanamycin (50 μg/ml), IPTG (0.5 M), and X-Gal (40 μg/ml). After 24 h of growth at 30 °C, interaction could be observed via blue colonies on the plates, while no interaction could be identified via white colonies. As a positive control, *E. coli* BTH101 cells were co-transformed with pUT18C-zip and pKT25-zip. For a negative control, *E. coli* BTH101 cells were co-transformed with the empty plasmids pUT18C and pKT25.

### Heterologous expression of *ccdB*-like in *E. coli*

*E. coli* Dh5α-λ*pir* cells were transformed with the pBAD24-ccdB-like plasmid. To induce gene expression, P_*ara*_ was activated by adding 0.2% arabinose to the medium.

### Bioinformatics analysis

Structure prediction of XreR2 and XreR1 was performed by Phyre2 [14] and visualized using USCF Chimera 1.13.1 (Resource for Biocomputing, Visualization, and Informatics). Additional domain prediction was performed by using InterPro (https://www.ebi.ac.uk/interpro/).

### Heterologous overproduction and purification of recombinant XreR1 and XreR2

*E. coli* BL21(DE3) pLysS harboring plasmid pET28-His-SUMO-XreR1 or pET28-His-SUMO-XreR1 was grown to exponential phase at 37 °C. Expression of genes encoding N-terminally His-SUMO-tagged XreR1 (His_6_-SUMO-XreR1) or XreR2 (His_6_-SUMO-XreR2) was induced with 0.5 mM isopropyl-β-D-thiogalactopyranoside (IPTG) and the bacteria were incubated at 18 °C over-night. Subsequently, the cells were harvested and washed with at 6.000 rpm for 30 min at 4 °C. The cell pellet was frozen in liquid nitrogen and stored at − 80 °C until further use. Cells were resuspended in 0.2 ml/g lysis buffer [50 mM Tris/HCl pH 7.5, 5% glycerol (v/v), 10 mM MgCl2, 0.5 mM phenylmethane sulfonyl fluoride (PMSF), 1 mM dithiotreitol (DTT), 10 ng/ml DNAse] and lysed by passage through a high-pressure cell disrupter (Constant Systems, Northants, UK). After centrifugation (1 h at 45.000 rpm and 4 °C) of the disrupted cells, the supernatant containing the respective cytosolic His_6_-SUMO-protein was incubated with Ni^2+^-nitrilotriacetic acid (NTA) resin (Qiagen, Hilden, Germany) preequilibrated with lysis buffer. After 1 h of incubation, the protein-resin complex was washed twice with washing buffer (50 mM Tris/HCl pH 7.5, 10% glycerol (v/v), 500 mM NaCl, 10 mM imidazole, 2 mM β-mercaptoethanol (MeOH)). Finally, the His-SUMO-tagged protein was eluted in several fractions with buffer containing 250 mM imidazole, 50 mM Tris/HCl pH 7.5, 10% glycerol (v/v), 500 mM NaCl, 2 mM β-MeOH. Both proteins were dialyzed against XreR protein buffer (50 mM Tris/HCl pH 7.5, 10% glycerol (v/v), 500 mM NaCl, 2 mM β-MeOH) over night at 4 °C. To cleave off the His-SUMO tag, 1 mg of the protease Senp2 per 500 mg protein was added to the respective dialysed His-SUMO-tagged protein and another 4 h step of dialysis against the XreR protein buffer was performed. Subsequently, the Ni^2+^-NTA based affinity chromatography was repeated. As the tag was separated from the protein, the protein eluted in the flow through while only the tag bound to the beads and were eventually eluted using elution buffer. Protein concentrations were determined using NanoDrop (ThermoFisher Scientific, Frankfurt, Germany).

### Surface plasmon resonance (SPR) spectroscopy

SPR analysis was performed in a Biacore 3000 (GE Healthcare, München, Germany) using carboxymethyl dextran sensor chips that were pre-coated with streptavidin (Sensor Chip SA, GE Heathcare, München, Germany). DNA fragments comprising the respective promoter regions were 5′-biotinylated via PCR using the primers Btn-PxreR2 fwd and PxreR2 rev for genomic Btn-P_*xreR2*_ amplification. Genomic Btn-P_*xreR1*_ was achieved using the primer pair Btn-PxreR1 fwd + PxreR1 rev. Lastly, Btn-P_*pTAS*_ was amplified using Btn-Ptas fwd and Ptas rev. Before immobilization of the DNA fragments, the chip was equilibrated by three 90 μl injections using 1 M NaCl/50 mM NaOH at a flow rate of 10 μl/min. 10 nM of the respective biotinylated promoter DNA was injected using a contact time of 420 s and a flow rate of 10 μl/min. Approximately, 600 RU of P_*xreR1*_ was captured onto flow cell 2, P_*xreR2*_ onto flow cell 3 and P_*pTAS*_ onto flow cell 4, respectively, of the chip. XreR2, XreR1 or a 50:50 mixture of both were diluted in dialysis buffer and passed over flow cells 1 to 4 in different concentrations XreR1 (0 nM, 1 nM, 2.5, 5 nM, 10 nM, 25 nM, 50 nM), XreR2 (100 nM, 250 nM, 500 nM, 1000 nM, 2000 nM) and XreR1:XreR2 (50 nM, 100 nM, 250 nM, 500 nM, 1000 nM) using a contact time of 180 s followed by a 300 s dissociation time before the next cycle started. The XreR1:XreR2 combination could not be tested at higher concentrations than 1000 nM since XreR1 could not be concentrated higher than 1 μM. The experiments were carried out at 25 °C at a flow rate of 30 μl/min. After each cycle, regeneration of the surface was achieved by injection of 2.5 M NaCl for 60 s followed by 0.5% (w/v) SDS for 60 s at 30 μ/min flow rate. Sensorgrams were recorded using the Biacore 3000 control software and analyzed with the BIAevaluation 4.1.1 software (GE Healthcare, München). The surface of flow cell 1 was used to obtain blank sensorgrams for subtraction of bulk refractive index background. The referenced sensorgrams were normalized to a baseline of 0. The 1:1 binding algorithm was used for calculation of the binding affinity.

## Data Availability

All data generated or analysed during this study are included in this published article or are available from the corresponding author on reasonable request.
